# *catena*-Poly[[bis­(nitrato-κ^2^ *O*,*O*′)cobalt(II)]-μ-4,4′-bis­(pyrazol-1-ylmeth­yl)biphenyl-κ^2^ *N* ^2^:*N* ^2′^]

**DOI:** 10.1107/S160053681001977X

**Published:** 2010-05-29

**Authors:** Jin-Sheng Gao, Xue Wang, Zhi-Yong Ding, Guang-Feng Hou

**Affiliations:** a College of Chemistry and Materials Science, Heilongjiang University, Harbin 150080, People’s Republic of China; b Engineering Research Center of Pesticide of Heilongjiang Province, Heilongjiang University, Harbin 150080, People’s Republic of China; c Daqing New Century Industrial Co. Ltd, Daqing 163511, People’s Republic of China

## Abstract

In the title compound, [Co(NO_3_)_2_(C_20_H_18_N_4_)]_*n*_, the Co^II^ atom lies on a crystallographic twofold axis and the coordination geometry can be considered as a slightly distorted tetra­hedron defined by two O atoms from two nitrate groups and two N atoms from two ligand mol­ecules. A distorted octa­hedron may be assumed when two of the symmetry-related nitrate O atoms with Co—O distances of 2.3449 (19) Å are added to the coordination environment. Another twofold axis, passing through the middle of the biphenyl bonds, is observed in the crystal structure. A chain is built up by the ligands linking the Co^II^ ions along [101].

## Related literature

For a related polymeric bis­(pyrazole) dinitratocobalt(II) structure, see: Chen *et al.* (1997 ▶). For the isotypic Zn structure, see: Wang *et al.* (2010 ▶). For the synthesis and structure of a three-dimensional polymeric Zn(II) network compound, see: Zhu *et al.* (2002 ▶).
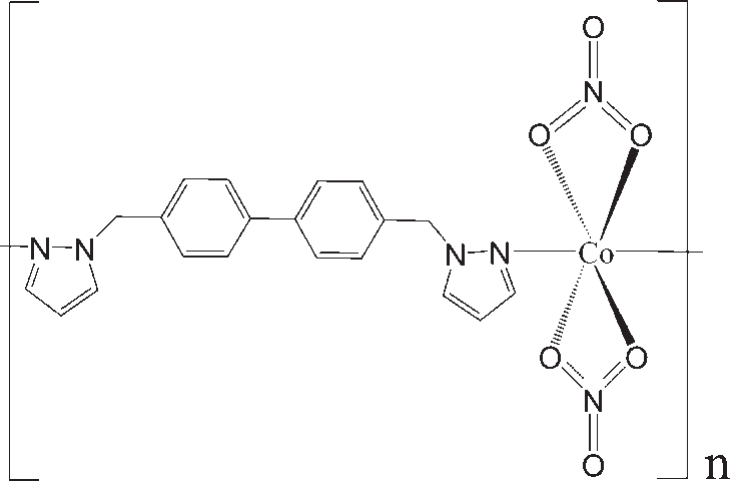


## Experimental

### 

#### Crystal data

[Co(NO_3_)_2_(C_20_H_18_N_4_)]
*M* *_r_* = 497.33
Monoclinic, 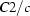

*a* = 14.133 (6) Å
*b* = 13.631 (8) Å
*c* = 10.806 (5) Å
β = 96.211 (18)°
*V* = 2069.5 (18) Å^3^
*Z* = 4
Mo *K*α radiation
μ = 0.88 mm^−1^
*T* = 291 K
0.24 × 0.23 × 0.21 mm

#### Data collection

Rigaku R-AXIS RAPID diffractometer
Absorption correction: multi-scan (*ABSCOR*; Higashi, 1995 ▶) *T*_min_ = 0.814, *T*_max_ = 0.834
9940 measured reflections
2346 independent reflections
1931 reflections with *I* > 2σ(*I*)
*R*_int_ = 0.043

#### Refinement

*R*[*F* ^2^ > 2σ(*F* ^2^)] = 0.036
*wR*(*F* ^2^) = 0.091
*S* = 1.07
2346 reflections
150 parameters
H-atom parameters constrained
Δρ_max_ = 0.39 e Å^−3^
Δρ_min_ = −0.31 e Å^−3^

### 

Data collection: *RAPID-AUTO* (Rigaku 1998 ▶); cell refinement: *RAPID-AUTO*; data reduction: *CrystalStructure* (Rigaku/MSC, 2002 ▶); program(s) used to solve structure: *SHELXS97* (Sheldrick, 2008 ▶); program(s) used to refine structure: *SHELXL97* (Sheldrick, 2008 ▶); molecular graphics: *SHELXTL* (Sheldrick, 2008 ▶); software used to prepare material for publication: *SHELXL97*.

## Supplementary Material

Crystal structure: contains datablocks I, global. DOI: 10.1107/S160053681001977X/si2260sup1.cif

Structure factors: contains datablocks I. DOI: 10.1107/S160053681001977X/si2260Isup2.hkl

Additional supplementary materials:  crystallographic information; 3D view; checkCIF report

## Figures and Tables

**Table 1:** Selected bond lengths (Å)

Co1—O1	2.0395 (18)
Co1—N1	2.0463 (18)
Co1—O2	2.3444 (19)

**Fig. 1.**
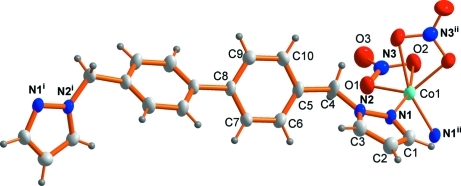
The molecular structure of title compound, showing the atom-labeling scheme and displacement ellipsoids drawn at the 50% probability level. Symmetry codes: i = -x + 1, y, -z - 1/2; ii = -x + 2, y, -z + 1/2.

**Fig. 2.**
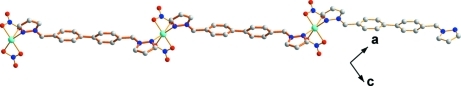
A section of the chain structure of the title compound extending along the [1 0 1] direction.

## supplementary crystallographic information

### Comment

The structures of the metal derivative 1,4-bis(pyrazole) benzene are known for zinc and cobalt (Chen *et al.*, 1997). In order to enrich the research of this kinds of ligand, a new ligand 4,4'-bis(pyrazole) biphenyl with longer spacer was synthesized. In here, we report the strucuture of the title compound, which is a isomorphic compound of our previous report (Wang *et al.*, 2010).

The central Co atom lies on a crystallographic twofold axis and the coordination geometry can be considered as a slightly distorted tetrahedron defined by two O atoms from two nitrate groups and two N atoms from two ligand molecules. A distorted octahedron may be assumed when two of the C_2_ related nitrate oxygen atoms with Co—O distances of 2.345 (2) Å are added to the coordination environment. Another twofold axis, passing through the middle of the biphenyl bonds, is observed in the crystal structure (Figure 1, Table 1).

A one dimensional chain is built up by the ligands linking the Co^II^ ions along the [1 0 1] direction (Figure 2).

### Experimental

The 4,4'-bis(pyrazole-1-ylmethyl) biphenyl was synthesized by the reaction of pyrazole and 4,4'-bis(chloro) bibenzene under alkaline condition (Zhu *et al.*, 2002). Cobalt(II) dinitrate hexahydrate (0.582 g, 2 mmol) and 4,4'-bis(pyrazole-1-ylmethyl) biphenyl (0.628 g, 2 mmol) were dissolved in ethanol (20 ml), purple block-shaped crystals separated from the filtered solution after several days.

### Refinement

H atoms bound to C atoms were placed in calculated positions and treated as riding on their parent atoms, with C—H = 0.93 Å (aromatic), C—H = 0.97 Å (methylene), and with *U*_iso_(H) = 1.2*U*_eq_(C).

### Crystal data

**Table:**

[Co(NO_3_)_2_(C_20_H_18_N_4_)]	*F*(000) = 1020
*M**_r_* = 497.33	*D*_x_ = 1.596 Mg m^−^^3^
Monoclinic, *C*2/*c*	Mo *K*α radiation, λ = 0.71073 Å
Hall symbol: -C 2yc	Cell parameters from 7566 reflections
*a* = 14.133 (6) Å	θ = 3.0–27.5°
*b* = 13.631 (8) Å	µ = 0.88 mm^−^^1^
*c* = 10.806 (5) Å	*T* = 291 K
β = 96.211 (18)°	Block, brown
*V* = 2069.5 (18) Å^3^	0.24 × 0.23 × 0.21 mm
*Z* = 4	

### Data collection

**Table:**

Rigaku R-AXIS RAPID diffractometer	2346 independent reflections
Radiation source: fine-focus sealed tube	1931 reflections with *I* > 2σ(*I*)
graphite	*R*_int_ = 0.043
ω scan	θ_max_ = 27.5°, θ_min_ = 3.0°
Absorption correction: multi-scan (*ABSCOR*; Higashi, 1995)	*h* = −18→18
*T*_min_ = 0.814, *T*_max_ = 0.834	*k* = −17→17
9940 measured reflections	*l* = −14→12

### Refinement

**Table:**

Refinement on *F*^2^	Primary atom site location: structure-invariant direct methods
Least-squares matrix: full	Secondary atom site location: difference Fourier map
*R*[*F*^2^ > 2σ(*F*^2^)] = 0.036	Hydrogen site location: inferred from neighbouring sites
*wR*(*F*^2^) = 0.091	H-atom parameters constrained
*S* = 1.07	*w* = 1/[σ^2^(*F*_o_^2^) + (0.045*P*)^2^ + 0.959*P*] where *P* = (*F*_o_^2^ + 2*F*_c_^2^)/3
2346 reflections	(Δ/σ)_max_ < 0.001
150 parameters	Δρ_max_ = 0.39 e Å^−^^3^
0 restraints	Δρ_min_ = −0.30 e Å^−^^3^

### Special details

**Table:**

Geometry. All e.s.d.'s (except the e.s.d. in the dihedral angle between two l.s. planes) are estimated using the full covariance matrix. The cell e.s.d.'s are taken into account individually in the estimation of e.s.d.'s in distances, angles and torsion angles; correlations between e.s.d.'s in cell parameters are only used when they are defined by crystal symmetry. An approximate (isotropic) treatment of cell e.s.d.'s is used for estimating e.s.d.'s involving l.s. planes.
Refinement. Refinement of *F*^2^ against ALL reflections. The weighted *R*-factor *wR* and goodness of fit *S* are based on *F*^2^, conventional *R*-factors *R* are based on *F*, with *F* set to zero for negative *F*^2^. The threshold expression of *F*^2^ > σ(*F*^2^) is used only for calculating *R*-factors(gt) *etc*. and is not relevant to the choice of reflections for refinement. *R*-factors based on *F*^2^ are statistically about twice as large as those based on *F*, and *R*- factors based on ALL data will be even larger.

### Fractional atomic coordinates and isotropic or equivalent isotropic displacement parameters (Å^2^)

**Table:**

	*x*	*y*	*z*	*U*_iso_*/*U*_eq_	
C1	0.88489 (15)	−0.14358 (16)	0.29942 (19)	0.0386 (5)	
H1	0.9333	−0.1663	0.3575	0.046*	
C2	0.79651 (15)	−0.18759 (17)	0.2749 (2)	0.0420 (5)	
H2	0.7746	−0.2436	0.3119	0.050*	
C3	0.74852 (14)	−0.13104 (15)	0.1848 (2)	0.0367 (5)	
H3	0.6867	−0.1416	0.1484	0.044*	
C4	0.78951 (16)	0.02164 (16)	0.0679 (2)	0.0449 (6)	
H4A	0.8434	0.0250	0.0195	0.054*	
H4B	0.7869	0.0830	0.1128	0.054*	
C5	0.69915 (14)	0.01146 (16)	−0.02073 (19)	0.0350 (5)	
C6	0.68227 (14)	−0.06955 (16)	−0.0965 (2)	0.0385 (5)	
H6	0.7242	−0.1223	−0.0887	0.046*	
C7	0.60311 (14)	−0.07260 (16)	−0.18418 (19)	0.0364 (5)	
H7	0.5914	−0.1284	−0.2330	0.044*	
C8	0.54120 (13)	0.00614 (15)	−0.20021 (17)	0.0301 (4)	
C9	0.55799 (14)	0.08674 (16)	−0.1228 (2)	0.0391 (5)	
H9	0.5169	0.1402	−0.1312	0.047*	
C10	0.63546 (14)	0.08820 (16)	−0.0331 (2)	0.0402 (5)	
H10	0.6447	0.1419	0.0199	0.048*	
Co1	1.0000	0.03534 (3)	0.2500	0.03214 (14)	
N1	0.89123 (11)	−0.06439 (12)	0.22882 (15)	0.0327 (4)	
N2	0.80555 (11)	−0.05793 (12)	0.15780 (15)	0.0304 (4)	
N3	1.06867 (12)	0.16070 (13)	0.10362 (17)	0.0382 (4)	
O1	1.00539 (10)	0.09498 (12)	0.07781 (14)	0.0456 (4)	
O2	1.10495 (11)	0.16308 (13)	0.21497 (15)	0.0497 (4)	
O3	1.09153 (13)	0.21630 (14)	0.02403 (17)	0.0627 (5)	

### Atomic displacement parameters (Å^2^)

**Table:**

	*U*^11^	*U*^22^	*U*^33^	*U*^12^	*U*^13^	*U*^23^
C1	0.0360 (11)	0.0383 (12)	0.0379 (11)	−0.0004 (9)	−0.0118 (9)	0.0046 (9)
C2	0.0409 (12)	0.0387 (12)	0.0445 (12)	−0.0066 (9)	−0.0036 (10)	0.0068 (9)
C3	0.0260 (9)	0.0406 (12)	0.0412 (11)	−0.0070 (8)	−0.0065 (8)	−0.0005 (9)
C4	0.0351 (11)	0.0422 (13)	0.0508 (13)	−0.0093 (9)	−0.0255 (10)	0.0112 (10)
C5	0.0261 (10)	0.0404 (12)	0.0349 (11)	−0.0042 (8)	−0.0127 (8)	0.0051 (9)
C6	0.0305 (10)	0.0395 (11)	0.0420 (12)	0.0085 (9)	−0.0115 (9)	0.0005 (9)
C7	0.0328 (10)	0.0365 (11)	0.0366 (11)	0.0047 (8)	−0.0120 (9)	−0.0068 (9)
C8	0.0213 (9)	0.0374 (11)	0.0292 (10)	−0.0002 (7)	−0.0088 (8)	0.0017 (8)
C9	0.0315 (10)	0.0393 (12)	0.0431 (12)	0.0081 (9)	−0.0116 (9)	−0.0053 (9)
C10	0.0372 (11)	0.0381 (12)	0.0412 (12)	0.0003 (9)	−0.0145 (9)	−0.0090 (9)
Co1	0.0226 (2)	0.0312 (2)	0.0390 (2)	0.000	−0.01331 (15)	0.000
N1	0.0240 (8)	0.0335 (9)	0.0366 (9)	−0.0004 (6)	−0.0146 (7)	0.0010 (7)
N2	0.0225 (7)	0.0329 (9)	0.0327 (8)	−0.0018 (6)	−0.0111 (6)	0.0002 (7)
N3	0.0337 (9)	0.0333 (9)	0.0462 (10)	−0.0007 (7)	−0.0020 (8)	−0.0011 (8)
O1	0.0419 (8)	0.0440 (9)	0.0471 (9)	−0.0118 (7)	−0.0125 (7)	0.0013 (7)
O2	0.0416 (8)	0.0530 (10)	0.0504 (9)	−0.0103 (7)	−0.0142 (7)	−0.0041 (8)
O3	0.0737 (12)	0.0549 (12)	0.0594 (11)	−0.0208 (9)	0.0064 (9)	0.0111 (9)

### Geometric parameters (Å, °)

**Table:**

C1—N1	1.330 (3)	C7—H7	0.9300
C1—C2	1.385 (3)	C8—C9	1.386 (3)
C1—H1	0.9300	C8—C8^i^	1.498 (3)
C2—C3	1.364 (3)	C9—C10	1.382 (3)
C2—H2	0.9300	C9—H9	0.9300
C3—N2	1.334 (3)	C10—H10	0.9300
C3—H3	0.9300	Co1—O1	2.0395 (18)
C4—N2	1.457 (3)	Co1—O1^ii^	2.0395 (18)
C4—C5	1.517 (3)	Co1—N1	2.0463 (18)
C4—H4A	0.9700	Co1—N1^ii^	2.0463 (18)
C4—H4B	0.9700	Co1—O2	2.3444 (19)
C5—C10	1.377 (3)	Co1—O2^ii^	2.3444 (19)
C5—C6	1.380 (3)	N1—N2	1.364 (2)
C6—C7	1.386 (3)	N3—O3	1.216 (2)
C6—H6	0.9300	N3—O2	1.256 (2)
C7—C8	1.384 (3)	N3—O1	1.275 (2)
			
N1—C1—C2	110.92 (18)	C5—C10—C9	121.14 (19)
N1—C1—H1	124.5	C5—C10—H10	119.4
C2—C1—H1	124.5	C9—C10—H10	119.4
C3—C2—C1	105.08 (19)	O1—Co1—O1^ii^	133.02 (10)
C3—C2—H2	127.5	O1—Co1—N1	105.33 (6)
C1—C2—H2	127.5	O1^ii^—Co1—N1	105.37 (7)
N2—C3—C2	108.24 (17)	O1—Co1—N1^ii^	105.37 (7)
N2—C3—H3	125.9	O1^ii^—Co1—N1^ii^	105.33 (6)
C2—C3—H3	125.9	N1—Co1—N1^ii^	96.74 (10)
N2—C4—C5	114.35 (17)	O1—Co1—O2	57.99 (6)
N2—C4—H4A	108.7	O1^ii^—Co1—O2	86.45 (7)
C5—C4—H4A	108.7	N1—Co1—O2	162.95 (6)
N2—C4—H4B	108.7	N1^ii^—Co1—O2	91.76 (8)
C5—C4—H4B	108.7	O1—Co1—O2^ii^	86.45 (7)
H4A—C4—H4B	107.6	O1^ii^—Co1—O2^ii^	57.99 (6)
C10—C5—C6	118.75 (18)	N1—Co1—O2^ii^	91.76 (8)
C10—C5—C4	119.24 (19)	N1^ii^—Co1—O2^ii^	162.95 (6)
C6—C5—C4	121.82 (19)	O2—Co1—O2^ii^	84.08 (10)
C5—C6—C7	120.29 (19)	C1—N1—N2	105.32 (15)
C5—C6—H6	119.9	C1—N1—Co1	124.84 (13)
C7—C6—H6	119.9	N2—N1—Co1	129.07 (13)
C8—C7—C6	120.99 (19)	C3—N2—N1	110.44 (16)
C8—C7—H7	119.5	C3—N2—C4	130.38 (16)
C6—C7—H7	119.5	N1—N2—C4	119.18 (15)
C7—C8—C9	118.34 (17)	O3—N3—O2	123.31 (18)
C7—C8—C8^i^	121.46 (14)	O3—N3—O1	121.13 (18)
C9—C8—C8^i^	120.19 (13)	O2—N3—O1	115.56 (18)
C10—C9—C8	120.40 (19)	N3—O1—Co1	100.08 (12)
C10—C9—H9	119.8	N3—O2—Co1	86.31 (11)
C8—C9—H9	119.8		

Symmetry codes: (i) −*x*+1, *y*, −*z*−1/2; (ii) −*x*+2, *y*, −*z*+1/2.
